# Supplementary material for: The prevalence and risk of mortality associated with intradialytic hypertension among patients with end-stage kidney disease on haemodialysis: A systematic review and meta-analysis
Source: PLoS One. 2024 Jun 11;19(6):e0304633. doi: 10.1371/journal.pone.0304633 (PMC11166311; doi:10.1371/journal.pone.0304633)
Supplement: S2 Table — (DOCX) [file pone.0304633.s002.docx]

**Supplementary Table 2: Search Strategy on PubMed and EMBASE**

**PubMed**

| **Search No.** | **Query** | **Result** |
| --- | --- | --- |
| **3** | (#1) AND (#2) | *6,711* |
| **2** | (((intradialytic hypertension) OR (dialysis-dependent hypertension)) OR (intradialytic blood pressure elevation)) OR (dialysis hypertension) | *16,058* |
| **1** | ((((((prevalence) OR (frequency)) OR (incidence)) OR (occurrence)) OR (burden)) OR (preponderance)) OR (rate) | *7,256,594* |

**EMBASE**

| **Query** | **Result** |
| --- | --- |
| ("epidemiology"[MeSH Subheading] OR "epidemiology"[All Fields] OR "prevalence"[All Fields] OR "prevalence"[MeSH Terms] OR "prevalance"[All Fields] OR "prevalences"[All Fields] OR "prevalence s"[All Fields] OR "prevalent"[All Fields] OR "prevalently"[All Fields] OR "prevalents"[All Fields] OR ("epidemiology"[MeSH Subheading] OR "epidemiology"[All Fields] OR "frequency"[All Fields] OR "epidemiology"[MeSH Terms] OR "frequence"[All Fields] OR "frequences"[All Fields] OR "frequencies"[All Fields]) OR ("epidemiology"[MeSH Subheading] OR "epidemiology"[All Fields] OR "incidence"[All Fields] OR "incidence"[MeSH Terms] OR "incidences"[All Fields] OR "incident"[All Fields] OR "incidents"[All Fields]) OR ("epidemiology"[MeSH Subheading] OR "epidemiology"[All Fields] OR "occurrence"[All Fields] OR "epidemiology"[MeSH Terms] OR "occurrences"[All Fields]) OR ("burden"[All Fields] OR "burdened"[All Fields] OR "burdening"[All Fields] OR "burdens"[All Fields]) OR ("preponderance"[All Fields] OR "preponderances"[All Fields] OR "preponderant"[All Fields]) OR ("j rehabil assist technol eng"[Journal] OR "rate"[All Fields])) AND ((("intradialytic"[All Fields] OR "intradialytically"[All Fields]) AND ("hypertense"[All Fields] OR "hypertension"[MeSH Terms] OR "hypertension"[All Fields] OR "hypertension s"[All Fields] OR "hypertensions"[All Fields] OR "hypertensive"[All Fields] OR "hypertensive s"[All Fields] OR "hypertensives"[All Fields])) OR ("dialysis-dependent"[All Fields] AND ("hypertense"[All Fields] OR "hypertension"[MeSH Terms] OR "hypertension"[All Fields] OR "hypertension s"[All Fields] OR "hypertensions"[All Fields] OR "hypertensive"[All Fields] OR "hypertensive s"[All Fields] OR "hypertensives"[All Fields])) OR (("intradialytic"[All Fields] OR "intradialytically"[All Fields]) AND ("blood pressure"[MeSH Terms] OR ("blood"[All Fields] AND "pressure"[All Fields]) OR "blood pressure"[All Fields] OR "blood pressure determination"[MeSH Terms] OR ("blood"[All Fields] AND "pressure"[All Fields] AND "determination"[All Fields]) OR "blood pressure determination"[All Fields] OR ("blood"[All Fields] AND "pressure"[All Fields]) OR "blood pressure"[All Fields] OR "arterial pressure"[MeSH Terms] OR ("arterial"[All Fields] AND "pressure"[All Fields]) OR "arterial pressure"[All Fields] OR ("blood"[All Fields] AND "pressure"[All Fields])) AND ("elevate"[All Fields] OR "elevated"[All Fields] OR "elevates"[All Fields] OR "elevating"[All Fields] OR "elevation"[All Fields] OR "elevational"[All Fields] OR "elevations"[All Fields])) OR (("dialysance"[All Fields] OR "dialysances"[All Fields] OR "dialysation"[All Fields] OR "dialysator"[All Fields] OR "dialysators"[All Fields] OR "dialyse"[All Fields] OR "dialysed"[All Fields] OR "dialyser"[All Fields] OR "dialysers"[All Fields] OR "dialysing"[All Fields] OR "dialysis solutions"[Pharmacological Action] OR "dialysis solutions"[MeSH Terms] OR ("dialysis"[All Fields] AND "solutions"[All Fields]) OR "dialysis solutions"[All Fields] OR "dialysate"[All Fields] OR "dialysates"[All Fields] OR "dialyzate"[All Fields] OR "dialyzates"[All Fields] OR "dialysis"[MeSH Terms] OR "dialysis"[All Fields] OR "dialyses"[All Fields] OR "dialyzability"[All Fields] OR "dialyzable"[All Fields] OR "dialyzation"[All Fields] OR "dialyze"[All Fields] OR "dialyzed"[All Fields] OR "dialyzer"[All Fields] OR "dialyzer s"[All Fields] OR "dialyzers"[All Fields] OR "dialyzing"[All Fields] OR "renal dialysis"[MeSH Terms] OR ("renal"[All Fields] AND "dialysis"[All Fields]) OR "renal dialysis"[All Fields]) AND ("hypertense"[All Fields] OR "hypertension"[MeSH Terms] OR "hypertension"[All Fields] OR "hypertension s"[All Fields] OR "hypertensions"[All Fields] OR "hypertensive"[All Fields] OR "hypertensive s"[All Fields] OR "hypertensives"[All Fields]))) | *16,000* |
