# Supplementary material for: The prevalence and risk of mortality associated with intradialytic hypertension among patients with end-stage kidney disease on haemodialysis: A systematic review and meta-analysis
Source: PLoS One. 2024 Jun 11;19(6):e0304633. doi: 10.1371/journal.pone.0304633 (PMC11166311; doi:10.1371/journal.pone.0304633)
Supplement: S3 Table — (DOCX) [file pone.0304633.s003.docx]

**Supplementary Table 3: IDHTN Definition Criteria**

| **Category** | **Definition** | **References** |
| --- | --- | --- |
| **A** | Increase of SBP by 10mmHg or more during or at the end of the hemodialysis session | [3, 12, 13, 24, 25, 31, 32, 34, 36, 37, 39, 40, 41, 42, 44, 46, 48] |
| **B** | Increased SBP by 10mmHg or more in 3 consecutive hemodialysis sessions | [27, 35] |
| **C** | Increased SBP by 10mmHg or more in at least 4 out of 6 hemodialysis sessions | [23, 28, 33, 47, 49, 50] |
| **D** | Increased SBP by 10mmHg or increased mean arterial blood pressure (MABP) by 15mmHg during or at the end of the dialysis session | [26, 27, 38] |
| **D** | Increased MABP by 15mmHg or more during or at the end of hemodialysis | [30] |
| **E** | Increased SBP by 10mmHg or more in 3 consecutive HD sessions or any increase in blood pressure in the second or third hour of dialysis | [45] |
| **E** | Any increase in blood pressure during the second or third hour of hemodialysis or any increase in blood pressure resistant to ultrafiltration | [6] |
| **E** | Any increase in blood pressure in the second or third hour of hemodialysis | [43] |
