# Supplementary figures and images for: The prevalence and risk of mortality associated with intradialytic hypertension among patients with end-stage kidney disease on haemodialysis: A systematic review and meta-analysis
Source: PLoS One. 2024 Jun 11;19(6):e0304633. doi: 10.1371/journal.pone.0304633 (PMC11166311; doi:10.1371/journal.pone.0304633)

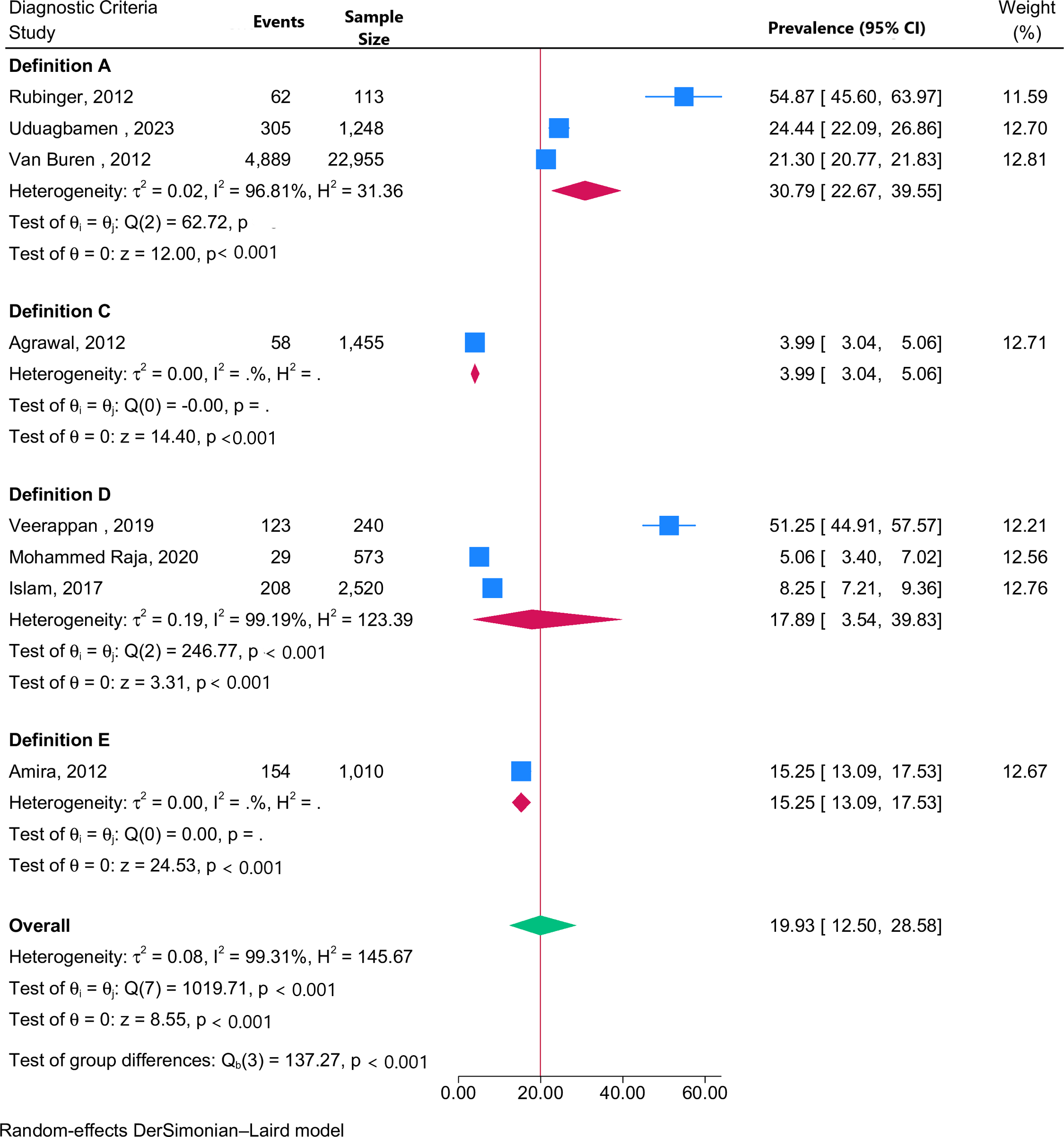

Supplement: S1 Fig — (TIFF) [file pone.0304633.s004.tiff]

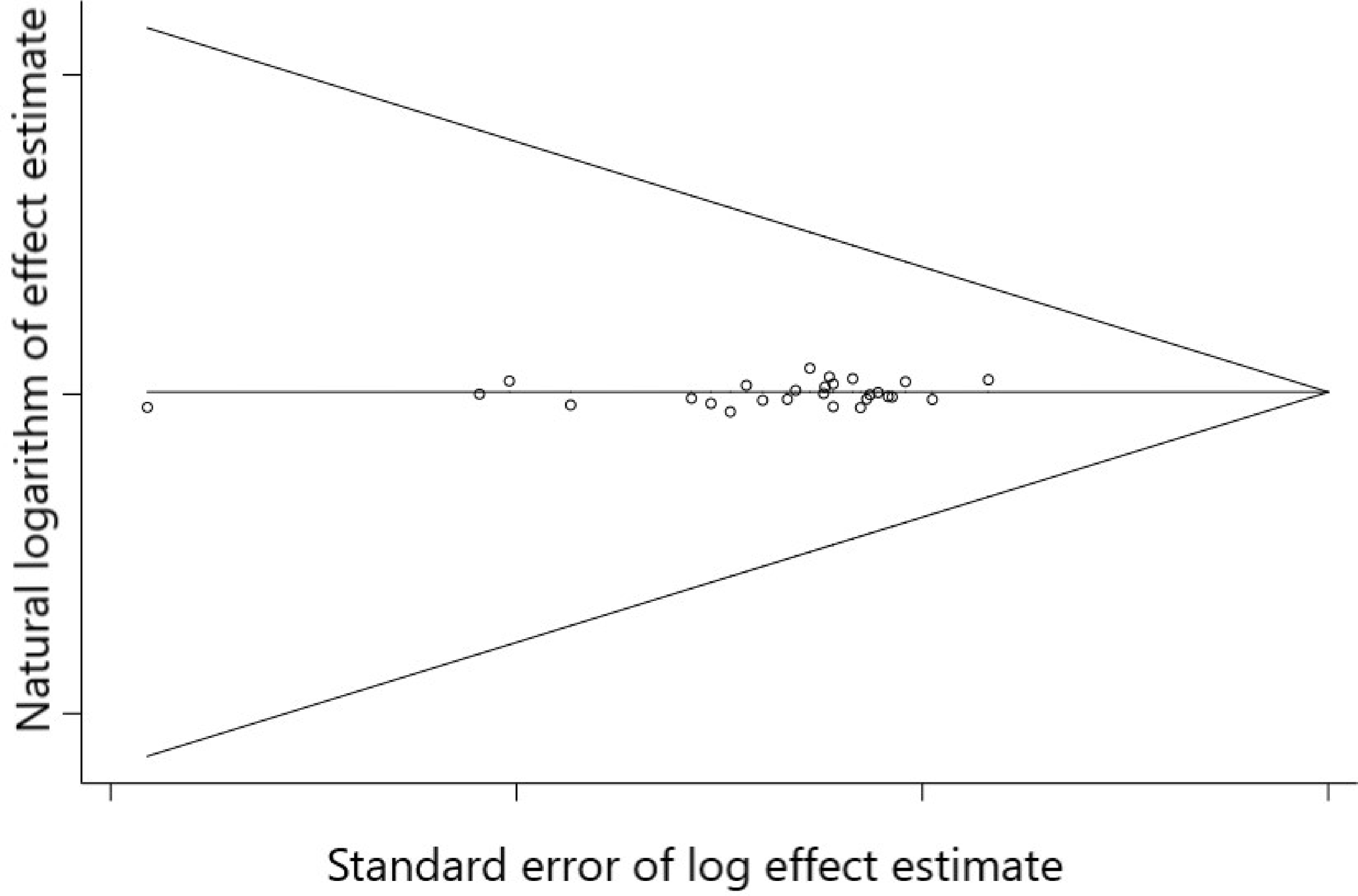

Supplement: S2 Fig — (TIF) [file pone.0304633.s005.tif]

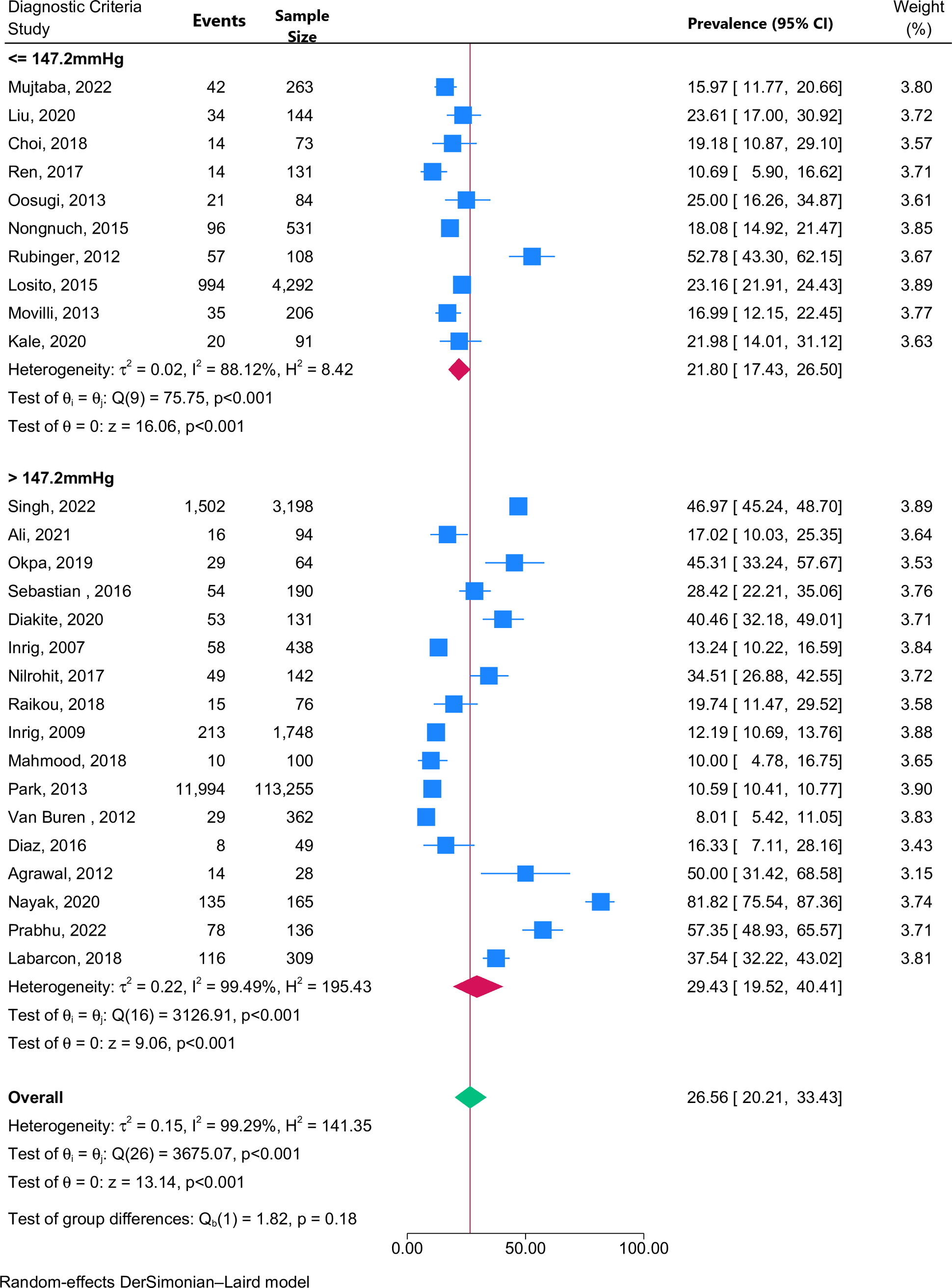

Supplement: S3 Fig — (TIFF) [file pone.0304633.s006.tiff]

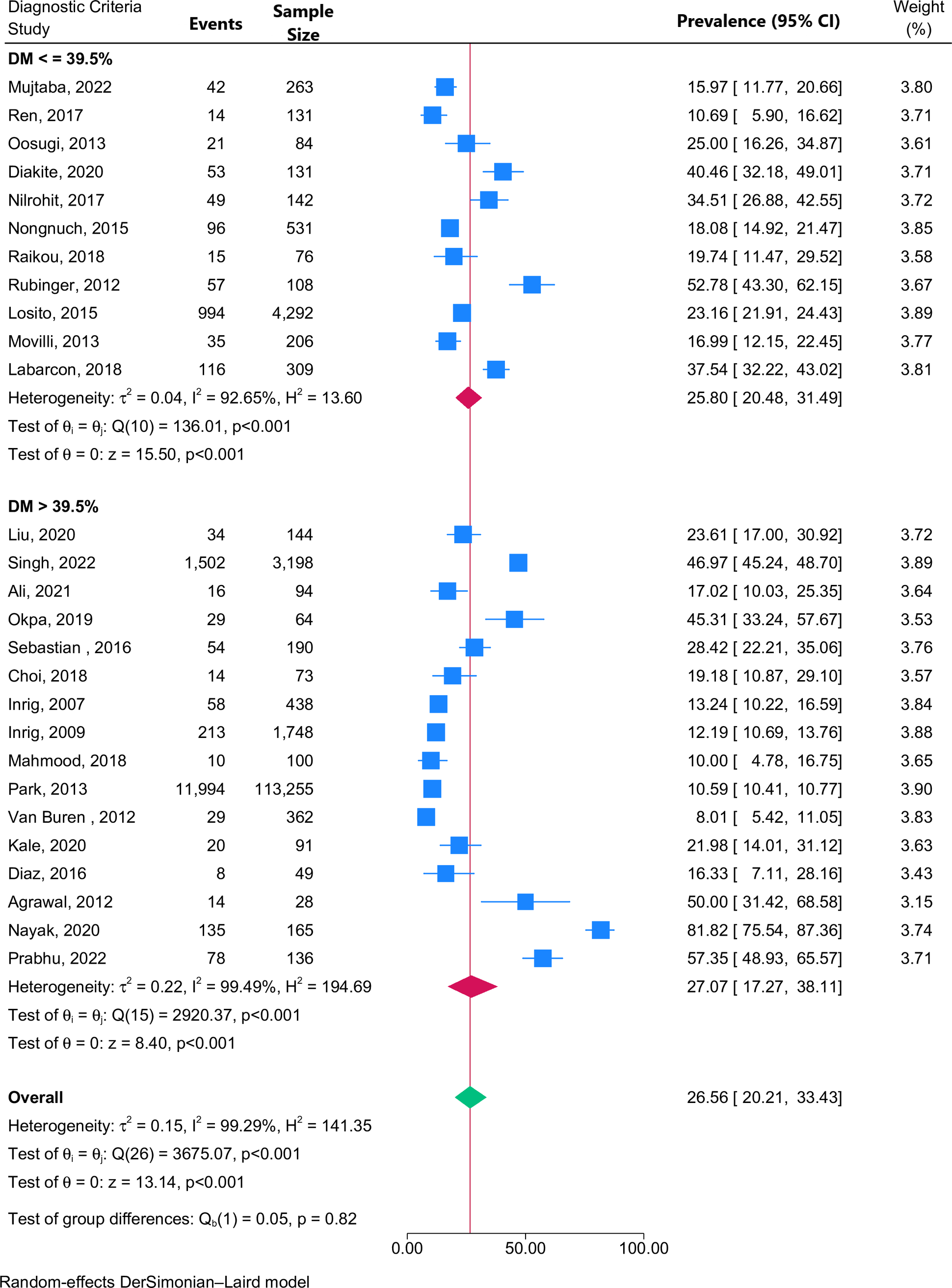

Supplement: S4 Fig — (TIFF) [file pone.0304633.s007.tiff]

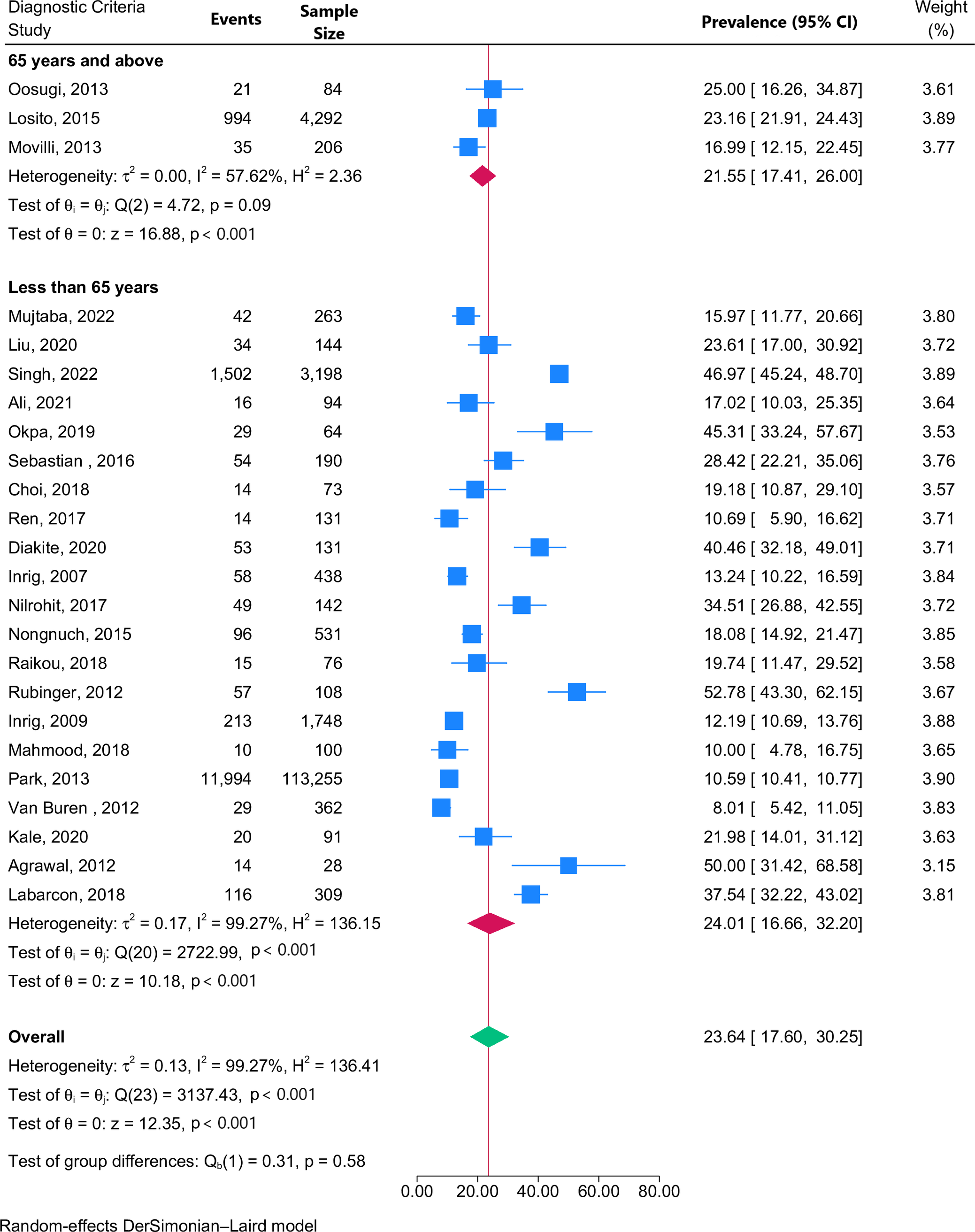

Supplement: S5 Fig — (TIFF) [file pone.0304633.s008.tiff]

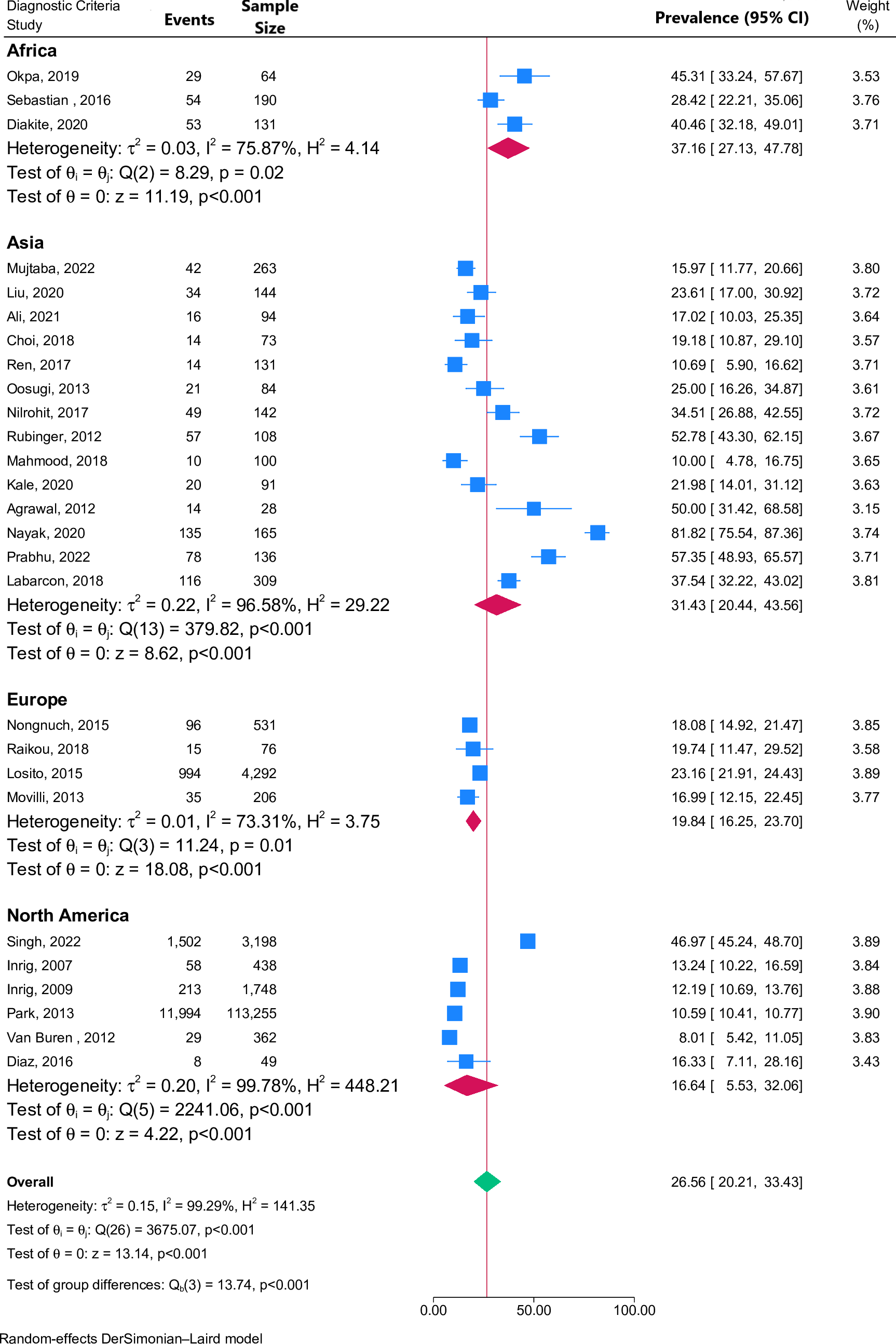

Supplement: S6 Fig — (TIFF) [file pone.0304633.s009.tiff]

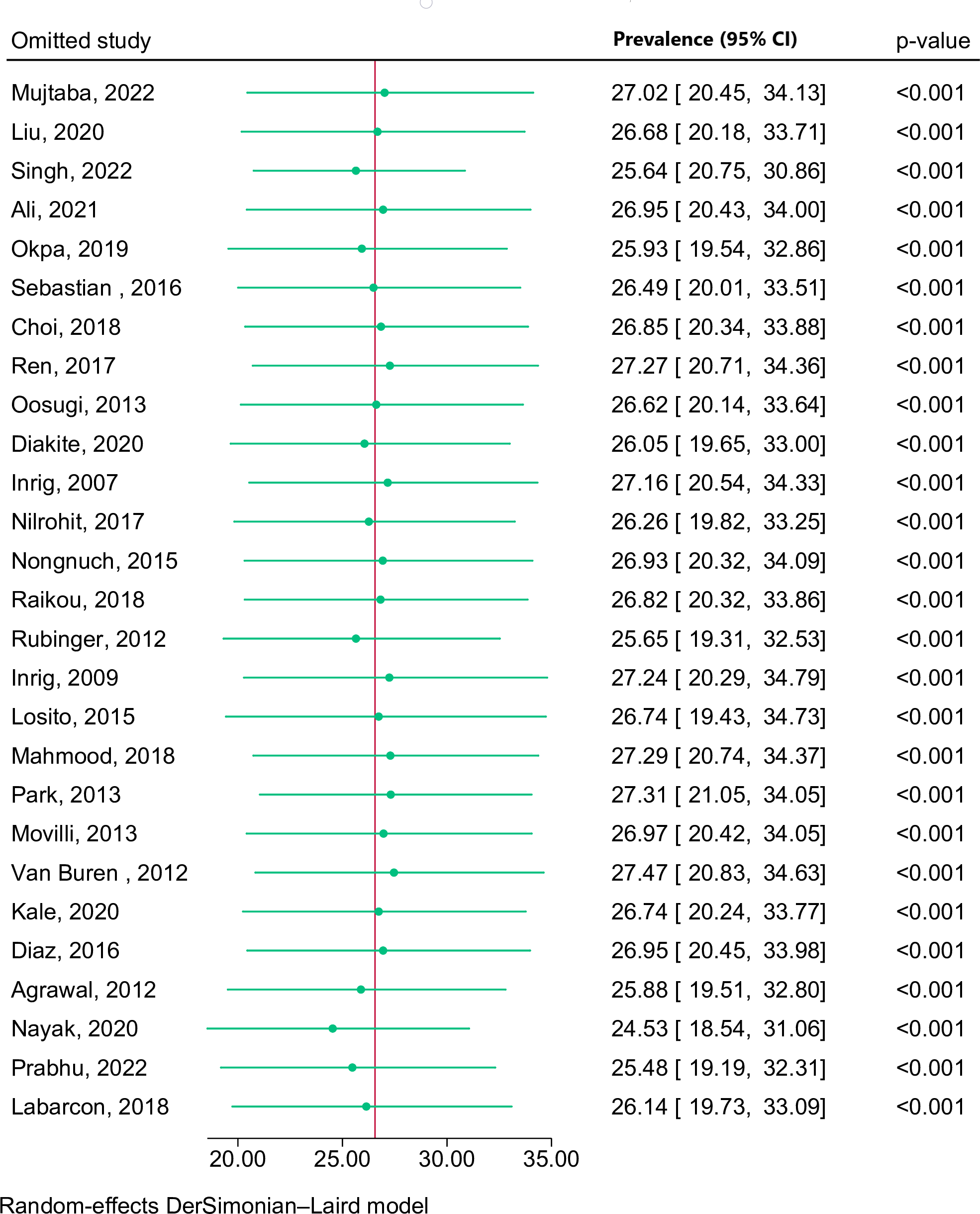

Supplement: S7 Fig — (TIFF) [file pone.0304633.s010.tiff]
