# Supplementary material for: The prevalence and risk of mortality associated with intradialytic hypertension among patients with end-stage kidney disease on haemodialysis: A systematic review and meta-analysis
Source: PLoS One. 2024 Jun 11;19(6):e0304633. doi: 10.1371/journal.pone.0304633 (PMC11166311; doi:10.1371/journal.pone.0304633)
Supplement: Attachment — Submitted filename: Response to Reviewers_IDHTN.docx [file pone.0304633.s011.docx]

|  | **Reviewer 1** |  |  |
| --- | --- | --- | --- |
| 1 | The different definitions of intradialytic hypertension are described in page 4, but the authors should add the A, B ,C…matches (it is stated in the supplementary file, but it is important to be clear in the main text too). | This has been included in the methodology. | Page 4, lines 94-100 |
| 2 | The fifth definition is suggested by KDIGO. This could be underlined and discussed a bit more.(less prevalence in this definition) | This observation is noted and has been included in the discussion. | Page 7, line 192-197 |
| 3 | The authors should explain why they use the median values of SBP /DBP and age as cut-off values (supplementary files). I believe that it is more clinically relevant to use eg in age >65 years old vs < 65years old | Thank you, again. We have re-categorized the age groups as suggested. Rerun the analysis and made the necessary changes in the result section. | Page 6, Lines 169-171. |
| 4 | Page 7. Ref 51 the correct form is Dorhout et al instead of Mee et al. | Thank you. This has been corrected. | Page 7, Line 186. |
|  |  |  |  |
|  | **Reviewer 2** |  |  |
| 1 | Please give the exact dates for your included studies. | The search of the articles were done between March and June, 2023. We included studies published between 1^st^ January 1990 and 30^th^ June 2023. | Page 4. Line 89-90 |
| 2 | please clarify what are the definitions A,B,C,D and E in the text | This has been included in the methodology. | P4 lines, line 98-103 |
| 3 | Refrain from comparing intradialytic hypertension and hypotension. This can't be the objective of this review | The discussion on Intradialytic hypotension has been deleted. |  |
| 4 | More detailed data is necessary. Please dissect more information from the studies for risk factors that result in intradalytic hypertension and discuss widely. The discussion of this review is still poor. If there's no data in the studies for risk factors, please add them as limitations. | We do not have data for the risk factors for IDHTN, hence we have included this as part of limitation. |  |
| 5 | Please revise the English language of your paper | The English language has been revised to improve its readability. |  |
